# Discovery, distribution and diversity of *Puroindoline-D1* genes in bread wheat from five countries (*Triticum aestivum* L.)

**DOI:** 10.1186/1471-2229-13-125

**Published:** 2013-09-08

**Authors:** Feng Chen, Huanhuan Li, Dangqun Cui

**Affiliations:** 1 Agronomy College, Henan Agricultural University, Zhengzhou 450002, China; 2 Key Laboratory of Physiological Ecology and Genetic Improvement of Food Crops in Henan Province, Zhengzhou 450002, China; 3 Collaborative Innovation Center of Henan Grain Crops, Zhengzhou 450002, China

**Keywords:** Bread wheat (*Triticum aestivum* L.), *Puroindoline-D1* genes, Grain texture, Primer walking, Functional marker

## Abstract

**Background:**

Grain texture is one of the most important characteristics in bread wheat (*Triticum aestivum* L.). *Puroindoline-D1* genes play the main role in controlling grain texture and are intimately associated with the milling and processing qualities in bread wheat.

**Results:**

A series of diagnostic molecular markers and dCAPS markers were used to characterize *Pina-D1* and *Pinb-D1* in 493 wheat cultivars from diverse geographic locations. A primer walking strategy was used to characterize PINA-null alleles at the DNA level. Results indicated that Chinese landraces encompassing 12 different *Puroindoline-D1* allelic combinations showed the highest diversity, while CIMMYT wheat cultivars containing 3 different *Puroindoline-D1* allelic combinations showed the lowest diversity amongst wheat cultivars from the five countries surveyed. Two novel *Pina-D1* alleles, designated *Pina-D1s* with a 4,422-bp deletion and *Pina-D1u* with a 6,460-bp deletion in the *Ha* (*Hardness*) locus, were characterized at the DNA level by a primer walking strategy, and corresponding molecular markers *Pina-N3* and *Pina-N4* were developed for straightforward identification of the *Pina-D1s* and *Pina-D1u* alleles. Analysis of the association of *Puroindoline-D1* alleles with grain texture indicated that wheat cultivars with *Pina-null/Pinb-null* allele, possessing an approximate 33-kb deletion in the *Ha* locus, have the highest SKCS hardness index amongst the different genotypes used in this study. Moreover, wheat cultivars with the PINA-null allele have significantly higher SKCS hardness index than those of *Pinb-D1b* and *Pinb-D1p* alleles.

**Conclusions:**

Molecular characterization of the *Puroindoline-D1* allele was investigated in bread wheat cultivars from five geographic regions, resulting in the discovery of two new alleles - *Pina-D1s* and *Pina-D1u*. Molecular markers were developed for both alleles. Analysis of the association of the *Puroindoline-D1* alleles with grain texture showed that cultivars with PINA-null allele possessed relatively high SKCS hardness index. This study can provide useful information for the improvement of wheat quality, as well as give a deeper understanding of the molecular and genetic processes controlling grain texture in bread wheat.

## Background

Grain texture is one of the most important characteristics determining the end-use properties of bread wheat (*Triticum aestivum* L.). It is well known that grain texture is mainly controlled by the *Ha* (*Hardness*) locus on the short arm of the 5D chromosome, even though the *Ha* loci were identified on homologous group 5 chromosome in bread wheat [1]. Compared with the *Ha* loci on the 5AS and 5BS chromosome, the *Ha* locus on the 5DS chromosome possesses three special genes - *Puroindoline a* (*Pina-D1*), *Puroindoline b* (*Pinb-D1*) and *Grain Softness Protein* (*Gsp-1*). *Puroindoline* genes have been proven to play a key role in modulating the grain texture in bread wheat [2-4]. However, the mechanism by which *Puroindoline* genes soften endosperm remains unknown. Moreover, the *Gsp-1* gene does not perform a significant function in determining grain texture [5,6].

The *Pina-D1* and *Pinb-D1* genes were shown to encode wheat endosperm-specific lipid binding proteins with a unique tryptophan-rich domain which was considered as being responsible for the strong affinity of the *Puroindoline-D1* protein to polar lipids. The *Puroindoline-D1* genes were identified in almost all of wheat and its diploid ancestors as well as related species, except the tetraploid *Triticum* species [7]. Both wild-type *Pina-D1* and *Pinb-D1* alleles produce a soft endosperm, whereas mutations in either *Pina-D1* or *Pinb-D1* results in endosperm hardening in bread wheat [8-10]. Since the first reported mutation in *Puroindoline-D1* genes was reported in bread wheat [2], many natural mutations in the *Pina-D1* and *Pinb-D1* genes have been found (i.e. *Pina-D1b* ~ *t* and *Pinb-D1b* ~ *ac* allele (see reviewed in [11-13])). All these mutations produce hard endosperm in bread wheat, and variations in *Puroindoline-D1* alleles have also been associated with differences in wheat quality [14-16]. In most geographic regions, bread wheat cultivars with the *Pinb-D1b* allele are predominant in bread wheat but there are some exceptions, e.g. the PINA null is the most popular allele in the CIMMYT (International Maize and Wheat Improvement Center) bread wheat cultivars [17] and *Pinb-D1p* is prevalent in the Chinese landrace cultivars [18,19]. Moreover, cultivars with the PINA-null allele tend to give harder endosperm than those with the *Pinb-D1b*[14,18,20-22], and the former may be less preferable from a milling standpoint. Recently, a novel group of *Pinb-2* variants described as *Pinb*-like genes [23-25] demonstrated to an inability to significantly contribute to grain softening when compared to the *Puroindoline-D1* genes [26,27] and also do not have intimate association with some quality characterizations surveyed by Mohler et al. [28].

Hexaploid wheat has a 29 kb smaller *Ha* locus in the D genome than its diploid donator *Ae. tauschii*, mainly due to transposable element insertions and two large deletions caused by illegitimate recombination (Chantret et al. [1]). It is possible, a large deletion in the *Pina-D1b* allele occurred after the DD genome of *Ae. tauschii* evolved into the AABBDD genomes of bread wheat. Compared with wild type, the *Pina-D1b* allele possesses a 15,380-bp deletion containing most of the *Pina-D1* coding region [29]. The *Pina-D1r* has a 10,415-bp deletion containing the entire *Pina* coding region and was identified in both Chinese and Japanese landraces [30,31]. Two corresponding molecular markers (*Pina-N1* and *Pina-N2*), which span the deletions, were developed for straightforward identification of the *Pina-D1b* and *Pina-D1r* alleles.

As the largest producer and consumer of bread wheat in the entire world, China is also a secondary origin center of bread wheat, holding a highly diverse stock of wheat germplasm. Of ten Chinese agro-ecological zones, the Yellow and Huai wheat production region covering eight provinces including all Henan is the largest and most important wheat production region, and accounts for 45% of the country’s total harvested area and 48% of the total wheat production [32]. Meanwhile, the Yellow and Huai valley is one of the secondary origin centers of bread wheat in China, and a large number of landraces have been collected or developed in this region, some of which have played an important role in improving wheat productivity. Moreover, in order to improve the genetic diversity and avoid germeplasm homogenization, a wide variety of international germplasms from abroad (e.g. CIMMYT, Australia, USA, and Europe) have been introduced or exchanged to this region for wheat breeding programs. The main purpose of this study was to investigate the distribution of *Puroindoline-D1* alleles in landraces and introduced cultivars of the Yellow and Huai Valley of China, in order to further characterize the molecular mechanism of PINA-null alleles, and to develop a robustly PCR-based molecular marker approach for wheat breeding.

## Methods

### Plant materials

In this study, a total of 493 bread wheat cultivars and advanced lines, including 204 Chinese landrace cultivars, 104 CIMMYT cultivars, 88 Australian cultivars, 53 Chilean cultivars and 44 cultivars from the Netherlands, were used to identify SKCS (Single Kernel Characterization System) hardness and *Puroindoline-D1* genes. Those cultivars or advanced lines, exchanged or introduced from different countries or regions by Seed Bank of Henan Agricultural University, possessed certain or multiple superior agronomic traits and are being popularly used as parents in wheat breeding programs in the Yellow and Huai Valleys of China. All the accessions surveyed in this study were planted at the Zhengzhou Scientific Research and Education Center of Henan Agricultural University during the 2009–10 and 2010–11 cropping seasons, and grew well under the local management practices which involved the use of a supporting net for Chinese landraces. We harvested them at the different stages according to the maturity of each accession to make sure each cultivar was fully mature.

Seven near-isogenic lines (NILs) with different *Puroindoline-D1* alleles (*Pina-D1b*, *Pinb-D1b*, *Pinb-D1c*, *Pinb-D1d*, *Pinb-D1e*, *Pinb-D1f* and *Pinb-D1g*), kindly provided by Prof. Xia Xianchun from Chinese Academy of Agricultural Sciences, were planted in Zhengzhou and Zhoukou in 2011–2012 cropping seasons, and were used to further examine the influence of *Puroindoline-D1* alleles on grain texture. These lines were developed at the USDA-ARS Western Wheat Quality Laboratory, Pullman, Washington. The NILs were developed by crossing donor parents possessing unique *Puroindoline a* and *Puroindoline b* gene haplotypes as male to the soft white spring wheat cultivar Alpowa. Seven backcrosses were conducted such that the general pedigree of each NIL is: Alpowa/donor parent//7*Alpowa [33].

The kernel hardness index of all wheat cultivars and advanced lines were measured by the Perten Single Kernel Characterization System (SKCS) 4100, following the manufacturer’s operation procedure (Perten Instruments North America Inc., Springfield, IL). The mean, standard deviation (SD), and distribution of SKCS hardness data, were used to classify the cultivars into ‘soft’, ‘mixed’, and ‘hard’ types.

### DNA extraction and PCR parameters

Genomic DNA of each hard wheat cultivar surveyed was separately extracted from three pulverized kernels following the method of Chen et al. [24]. Genomic DNA from seedlings was used for either marker development or primer walking strategy [18]. PCR amplifications were performed in a PTC-200 Peltier Thermocycler or an ABI 9700 and were conducted in 25 μl reactions using 100 ng of genomic DNA, 10 pmol of each primer, 200 μM of each dNTP mix, 1× *Taq* DNA polymerase reaction buffer with 1.5 μM of MgCl_2_, and 0.5 units of *Taq* DNA polymerase. The cycling conditions were 94°C for 5 min following 35 cycles of 94°C for 50 s, 50°C to 65°C for 50 s (primer-specific annealing temperatures, see Table 1), 72°C for 1 min, following a 10-min final extension time at 72°C. All PCR products were separated via gel-electrophoresis on a 1.5% agarose gel stained with ethidium bromide and visualized by UV light.

**Table 1:** **PCR primers used for generating *Puroindoline-D1 *alleles and primer walking**

**Primer name**	**Forward primer**	**Reverse primer**	**Tm**^**a**^	**Expected PCR size**
Pinb-D1b1	ATGAAGGCCCTCTTCCTCA	CTCATGCTCACAGCCGCT	58°С	250 bp
Pinb-D1b2	ATGAAGGCCCTCTTCCTCA	CTCATGCTCACAGCCGCC	58°С	250 bp
Pina-D1	CATCTATTCATCTCCACCTGC	GTGACAGTTTATTAGCTAGTC	58°С	524 bp
Pinb-D1	GAGCCTCAACCCATCTATTCATC	CAAGGGTGATTTTATTCATAG	58°С	597 bp
Pina-N1	AATACCACATGGTTCTAGATACTG	GCAATACAAAGGACCTCTAGATT	60°С	776 bp
Pina-N2	TCAACATTCGTGCATCATCA	CTTCATTCGTCAGAGTTCCAT	60°С	436 bp
Pina-N3	CATCTATTCATCTCCACCTGC	CACTATATTGCCGGGATTTT	58°С	440 bp
Pina-N4	AGTGGTCTGATGGAAGCGT	TGGAAAAAACTAGGTTGGGA	54°С	546
BsrDI_Pina-D1n	TCACCTGGCGTTGGTGGCAAT	CGGCAGGTTCTTGGCTTCTTGTAT	66°С	197
BalI_Pina-D1l	GAGTGTTGCAGTCGGCTTGG	GGCAGGTTCTTGGCTTCTTGT	61°С	143
Pina-1	TACCTGTAGCCCCCAAGTTT	GAGTCGCTGCAGGCTTACG	58°С	677 bp
Pina-2	AATGCCCAACCTATAACCCG	CCATAGCCATGCCTCTTGAT	57°С	647 bp
Pina-3	AACCAGACCGGGCTGATAGT	CCAAGACGATGGAGGGAAAG	62°С	889 bp
Pina-4	GATAACCCTAATCCGGAGTAA	CAAATCTTGCCAGTTTCAGC	54°С	1036 bp
Pina-5	ATGGCAACAGGTCCTCTTCG	ATAGCTCAATGGGCAGGCAC	60°С	579 bp
Pina-6	TGGAAGGTTGAGGTGACTGC	TTTTTATTGGCGTTCGACTG	57°С	512 bp
Pina-7	GCAGACGGCGGTTGATAGTA	GTGAGAAGAGGGCGAGGGAA	62°С	720 bp
Pina-8	GGTGGGCGTCAACTGTCGTG	ATGCTACCTTGCTTTGTCCTCC	62°С	606 bp
Pina-9	TGACCAGGGAGCGAATACG	TGACGAGTTTCACGCTTCCA	59°С	1143 bp
Pina-10	ATAATAGCACCGGAACGCA	AGTCTTTCTCTCAGCATACACG	54°С	1022 bp
Pina-11	GACCCGCATATAAGAAAACCGAT	TCCCACATGTTAGTGTCTGCAAAG	61°С	760 bp
Pina-12	CTTTTATGGCATTGTACATGGGGAG	TGTCAGTGTGTTTTGGTGCAGGTGG	65°С	950 bp
Pina-13	AGAAACATTTGACATGAACGAC	AGATGAGATGAGGCCACACC	54°С	751 bp
Pina-14	GTATGTTCTTGGTGGTGGTTT	TGGTCTGGGAGGATGAATAG	54°С	687 bp
Pina-15	CAGGACGAAATACGTTGAAA	TGGAAAAAACTAGGTTGGGA	54°С	1004 bp
Pina-16	AGAGAGTGCCCCAAAAGGTA	GGAAATCCCTTTGGTCAATG	56°С	623 bp
Pina-17	TGACCAAAGGGATTTCCGTA	AAGATGGAGATGATGATGCC	54°С	511 bp
Pina-18	CTTCAAGTTCCTCCAGACCT	CACTATATTGCCGGGATTTT	54°С	484 bp
Pina-19	TGTTGTCTGAGTCTTCCTGTTT	GCTCTGGTGGTTCGACTTCT	54°С	624 bp
Pina-20	TGAGAGGCCTTGACATTTCC	AACGATGTGTGTCAGGCGGT	57°С	962 bp
Pina-21	CTCGCCTTCTTCTTTTGTCTCG	CCAACGGACTCATCGGCTCA	61°С	890 bp
Pina-22	CGATGAGTCCGTTGGAGGTA	GAAGATGGCCTTTTCGATGC	58°С	906 bp
Pina-23	GGGTTTTTCTCTCAACTGGG	GAACAGTTTTTCACAATGGG	54°С	1114 bp
Pina-24	AGTAAACAGGCACTCTCGCT	CTGGTTTTTCCCTGTTTCAT	54°С	985 bp
Pina-25	CAGGGAAAAACCAGCAAAAC	GCAGGAAGTATGTCAAGCGT	55°С	1041 bp
Pinb-1	AGCGGGGTACTAGACAACAG	GATAAATACATACACCTGTCGTTC	53°С	950 bp
Pinb-2	CAAAAGCGACGGGCACAGAG	CTCTACTTTCCGGATGTTGCGA	62°С	757 bp
Pinb-3	ATCCTCTCCTTGTCACCCTG	GCTCACGCTTTAAGCTTTTG	56°С	527 bp
Pinb-4	CAGAAAACCACGGCTAGAAG	GGACATTGTTGAGAACCACCT	55	529
Pinb-5	CTGCGGAAAAAAAAATCTGG	CTAACATCTAAAGCCGGAGG	55	1531
Pinb-6	AGTGCGTCAGACCGGTTTGT	GGTGGTGGTGATTGGTGAAG	58	1129
Pinb-7	GGAAATTGTGTCGCCTCATC	AAAGCCGCATCTTCTTGTAG	55	942
Pinb-8	CAGAGACGTGTTTATGGGAG	GCCCTTGTTGTCTTCTTTTA	52	925
Pinb-9	TTACTCGAAGGACTCGGAAG	AGAGATAGTGTTGGCATGGA	52	1201
Pinb-10	TGCCAACACTATCTCTGCCTC	ATGATCCCGTGACTAACTCCT	55	1115
Pinb-11	ACGGGTATCTCTGAAAGTGTC	TAAGCGTACGTGTAAGGTCG	53	1458
Pinb-12	AGTCTTATCTTGTTTCGGCG	CTCATATGCTCCATGTTTCTC	52	1501
Pinb-13	TGTTCGAGGAGCTGAAAATG	TGGTTCGCACGTGTCAAAT	56	1126

^a^ Tm = PCR annealing temperature.

### Genotyping of *Puroindoline-D1* alleles in bread wheat

Five soft wheat cultivars were randomly selected for directly sequencing their *Pina* and *Pinb* genes with primer sets Pina-D and Pinb-D (Table 1) because they all should be wild-type *Puroindoline-D1* genes, i.e. *Pina-D1a/Pinb-D1a*. We removed all mixed wheat cultivars from this study as they possibly contained more than one genotype for each cultivar [17,34].

For the hard wheat cultivars based on SKCS classification, we first divided them into three groups by amplifications with primer sets Pina-D (containing the whole *Pina-D1* coding region) and Pinb-D (containing whole *Pinb-D1* coding region) (Table 1), i.e. Group I with both expected fragments of *Pina-D1* and *Pinb-D1*, Group II with only expected *Pinb-D1* fragment and Group III without any expected fragment of *Pina-D1* and *Pinb-D1* genes. In Group I, the *Pinb-D1b* allele was initially identified by a reciprocal pair of primer sets Pinb-D1b1 and Pinb-D1b2 [3]. PCR products amplified with the Pinb-D primer set were digested by restriction enzymes *PvuII* and *Pf1MI* for identification of the *Pinb-D1c* and *Pinb-D1p* alleles, respectively, following the methods of Lillemo and Morris [9] and Li et al. [19]. For the other remaining cultivars in this group, the PCR products amplified with the Pina-D and Pinb-D primer sets were directly sequenced from both strands by SinoGenoMax Co., Ltd (http://www.sinogenomax.com/) and genotypes were confirmed by alignment with either known *Puroindoline-D1* alleles or the NCBI blast website (http://blast.ncbi.nlm.nih.gov/).

In Groups II and III, the Pina-N1 and Pina-N2 markers we previously developed [29,30] were firstly used to identify *Pina-D1b* and *Pina-D1r* alleles, respectively, and other remaining cultivars were used for the primer walking strategy illustrated below.

### Development of dCAPS marker

Although the *Pina-D1l* and *Pina-D1n* alleles were discovered in previous studies [8,18,30], no valid detection marker existed. This promoted us to establish a dCAPS (derived Cleaved Amplified Polymorphic Sequences) technique as a detection method. Two sets of primers for amplifying fragments containing SNPs in cultivars with the *Pina-D1l* or *Pina-D1n* allele were designed using the dCAPS Finder 2.0 (http://helix.wustl.edu/dcaps/dcaps.html) software along with the appropriate restriction enzymes [35]. The restriction enzymes *BalI* and *BsrDI* were used to directly digest the PCR products amplified with primer sets of BalI_Pina-D1l and BsrDI_Pina-D1n (Table 1), respectively, for the detection of *Pina-D1l* and *Pina-D1n* alleles. Cultivars with 176-bp and 124-bp digested fragments belong to *Pina-D1n* and *Pina-D1l* alleles, respectively.

### Primer walking strategy

**Figure 1:**
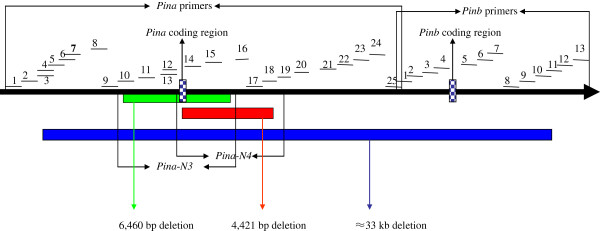
Schematic diagram of the primer walking strategy for illustrating the molecular characterization of PINA-null allele on the DNA level.

**Figure 2:**
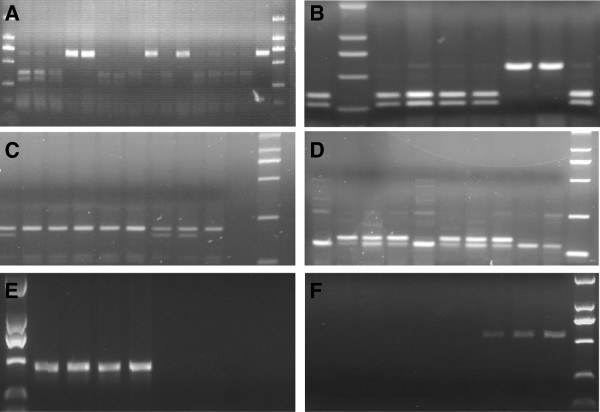
**Identification of six *Puroindoline-D1 *alleles by molecular markers. A**: Digestion of restriction enzyme *PvuII* for detection of *Pinb-D1c*; **B**: Digestion of restriction enzyme *Pf1MI* for detection of *Pina-D1p*; **C**: A dCAPS marker *BsrDI*_*Pina-D1n* for detection of *Pina-D1n*; **D**: A dCAPS marker *BalI_Pina-D1l* for detection of *Pina-D1l*; **E**: A new marker *Pina-N3* for detection of *Pina-D1s*; **F**: A new marker *Pina-N4* for detection of *Pina-D1u*.

Sequences of the *Ha-5D* loci (CT009735) from NCBI were used to design genome-specific primers around the *Pina* and *Pinb* coding regions again *Ha-5A* (CT009586) and *Ha-5B* (CT009585) loci. A total of 38 pairs of primer sets spanning an approximately 40-kb region (Table 1) were designed between −10,386 bp (reference to the ATG of the *Pina* gene) and +11,447 bp (reference to the ATG of the *Pinb* gene) for the primer walking strategy in order to illustrate the molecular mechanism of cultivars with the absence of the *Pina* gene or both *Pina* and *Pinb* genes (Figure 1). Based on the failure or success of PCR amplification, the size and position of each deletion fragment was deduced and new primers spanning estimated deletion fragment were designed for straightforward amplification of pending test samples (see schematic diagram in Figure 2). PCR products with successful amplification were sequenced to obtain the exact size and position of deletions.

### Sequencing and alignments of sequences

The targeted fragments were purified from the gels using Quick DNA Extraction Kit^™^ (Takara, http://www.takara.com.cn/). The products were then ligated into the pGEM-T Easy vector and transformed into *Escherchia coli* DH-5*α* strain. Plasmids with targeted fragments detected by Colony PCR were extracted by the Plasmid Rapid Isolation Kit^™^ (Biodev-tech Company, http://biodevtech.tech-food.com/). Five clones for each sample were sequenced from both strands by SinoGenoMax Co., Ltd.

Analysis and multiple alignments of sequences were performed with DNAMAN Version 6.0 and graphic data were analyzed to check sequencing results reliability with Chromas Version 1.4.5 and FinchTV version 1.4.0.

### Statistical analysis

SPSS (Statistical Product and Service Solutions) 19 software and LSR (least significant ranges) multiple comparison as well as Excel 2003 software were used to calculate the average and standard deviation of the SKCS hardness index, and to examine statistical significance among various *Puroindoline-Dl* alleles.

## Results

### Distribution of *Puroindoline-D1* alleles in bread wheat cultivars of five countries

Based on SKCS hardness index and distribution, 139 and 354 of the surveyed 493 wheat cultivars and advanced lines were classified as soft and hard genotypes, respectively. The 139 soft wheat cultivars were assumed to possess the wild type *Puroindoline-D1* haplotype (*Pina-D1a/Pinb-D1a*) [4,11-13]. This suggests that hard wheat is predominant in surveyed Chinese landraces and cultivars of Mexico, Australia, Chile and Netherlands even though soft wheat also possesses high distribution, with a percentage of 39%, in Chinese landraces surveyed.

All 354 hard wheat in this study were genotyped for *Puroindoline-D1* alleles. The results from PCR amplification of allele-specific primers, digestions with restriction enzymes *PvuII* and *Pf1MI* and sequencing indicated that 105, 4 and 47 of 354 hard wheat cultivars possessed the *Pinb-D1b*, *Pinb-D1c* and *Pinb-D1p* alleles (Figure 2A, 2B), respectively. Based on detection of molecular markers we developed previously [29,30], 129 and 21 cultivars belong to *Pina-D1b* and *Pina-D1r* alleles, respectively. For the remaining 48 cultivars, we tried to amplify the coding regions of *Pina* and *Pinb* genes with Primer set Pina-D and Pinb-D (Table 1). Then we sequenced all the *Pina* and *Pinb* genes of the 15 cultivars with entire amplifying fragments. Results of alignment with reported sequences of *Puroindoline-Dl* alleles showed that 3, 4, 6, 1 and 1 cultivars possessed *Pina-D1l*, *Pina-D1n*, *Pinb-D1d*, *Pinb-D1q* and *Pinb-D1t* alleles, respectively. Two new dCAPS markers were developed for identifying *Pina-D1l* and *Pina-D1n* alleles (Figure 2C, 2D), respectively, in order to efficiently detect them in the future because they are frequently present in Chinese landraces. Restriction enzymes *BalI* and *BsrDI* were used to digest the PCR products amplified with primer sets *BalI*-Pina-D1l and *BsrDI*-Pina-D1n (Table 1) for detection of *Pina-D1l* and *Pinb-D1n* alleles, respectively.

### Discovery of novel *Puroindoline-D1* alleles

In order to further illustrate the molecular mechanism of the remaining 33 cultivars with an absence of either *Pina* or *Pina* &*Pinb*, a series of primers (Table 1) were designed around the coding region of *Pina* and *Pinb* genes. Based on the primer walking strategy, we found that amplicons could be obtained with primer sets through Pina-1 to Pina-13 and Pina-19 to Pina-25 whereas no expected fragment was amplified with primer sets through Pina-14 to Pina-18 in two Chinese landrace cultivars Dahongmang and Hulutou (Table 2). A new molecular marker *Pina-N3* spanning deletion fragment was developed for identifying this new allele and then a 440-bp fragment was successfully amplified in those two cultivars (Figure 2E). The sequencing results of the 440-bp fragment indicated that there is a 4,422-bp deletion from +371 to 4792 bp (reference to ATG of *Pina*) in landraces Dahongmang and Hulutou compared with *Ha* sequence on the chromosome of 5DS in Chinese Spring (NCBI No: CT009735). This mutation with a single 4,422 bp deletion of *Pina* gene could be designated as *Pina-D1s* (Table 3) according to the nomenclature of 2007 Supplement of the Wheat Gene Catalogue [36,37], Morris and Behave [13] and Behave and Morris [11,12]. Furthermore, the new marker *Pina-N3* was used to identify the *Pina-D1s* allele in other cultivars and the results revealed that 28 Chinese landrace cultivars possess the *Pina-D1s* allele, suggesting that the *Pina-D1s* is prevalent in hard wheat of the Chinese landrace cultivars surveyed.

**Table 2:** Locations and amplifications of primers in different cultivars with PINA-null allele

**Primer**	**Position (Reference to ATG of *Pina *or *Pinb*)**		**Expected PCR product amplified**				
	**Forward primer**	**Reverse primer**	**Chinese Spring**	**Yunong 202**	**Dahongmang/Hulutou**	**Zuantoubaike**	**Yumai/Changyaomai/Yangqingke**
Pina-1	−10386^a^	−9170	Yes^c^	Yes	Yes	Yes	Yes
Pina-2	−9189	−8543	Yes	Yes	Yes	Yes	Yes
Pina-3	−8951	−8062	Yes	Yes	Yes	Yes	Yes
Pina-4	−9065	−8030	Yes	Yes	Yes	Yes	Yes
Pina-5	−8332	−7754	Yes	Yes	Yes	Yes	No
Pina-6	−7896	−7385	Yes	Yes	Yes	Yes	No
Pina-7	−7748	−7028	Yes	Yes	Yes	Yes	No
Pina-8	−6044	−5438	Yes	Yes	Yes	Yes	No
Pina-9	−5850	−4707	Yes	Yes	Yes	Yes	No
Pina-10	−4168	−3146	Yes	Yes	Yes	No	No
Pina-11	−2225	−1625	Yes	Yes	Yes	No	No
Pina-12	−950	0	Yes	Yes	Yes	No	No
Pina-13	−781	−30	Yes	Yes	Yes	No	No
Pina-D	−40	+474	Yes	Yes	No	No	No
Pina-14	+654	+1340	Yes	Yes	No	No	No
Pina-15	+1116	+2118	Yes	Yes	No	No	No
Pina-16	+3229	+3851	Yes	Yes	No	Yes	No
Pina-17	+3835	+4346	Yes	Yes	No	Yes	No
Pina-18	4283	+4821	Yes	Yes	No	Yes	No
Pina-19	+5116	+5740	Yes	Yes	Yes	Yes	No
Pina-20	+8439	+9400	Yes	Yes	Yes	Yes	No
Pina-21	+10695	+11585	Yes	Yes	Yes	Yes	No
Pina-22	+11560	+12466	Yes	Yes	Yes	Yes	No
Pina-23	+12260	+13373	Yes	Yes	Yes	Yes	No
Pina-24	+13464	+14448	Yes	Yes	Yes	Yes	No
Pina-25	+14435	+15475	Yes	Yes	Yes	Yes	No
Pinb-1	+15380 (−2766^b^)	+16330 (−1816)	Yes	Yes	Yes	Yes	No
Pinb-2	+16630 (−1516)	+17387 (−759)	Yes	Yes	Yes	Yes	No
Pinb-3	(−779)	(−253)	Yes	Yes	-^d^	-	No
Pinb-4	(−407)	(+121)	Yes	Yes	-	-	No
Pinb-D	(−52)	(+542)	Yes	Yes	-	-	No
Pinb-5	(+1024)	(+2554)	No	No	-	-	No
Pinb-6	(+2484)	(+3612)	Yes	Yes	-	-	No
Pinb-7	(+3539)	(+4480)	Yes	Yes	-	-	No
Pinb-8	(+4425)	(+5353)	Yes	Yes	-	-	No
Pinb-9	(+5275)	(+6475)	Yes	Yes	-	-	No
Pinb-10	(+6460)	(+7574)	Yes	Yes	-	-	Yes
Pinb-11	(+7427)	(+8884)	Yes	Yes	-	-	Yes
Pinb-12	(+8819)	(+10319)	Yes	Yes	-	-	Yes
Pinb-13	(+10321)	(+11447)	Yes	Yes	-	-	Yes

^a^ and ^b^ indicate relative positions of primers referenced to ATG in the *Pina* and *Pinb* genes, respectively.

^c^ “Yes” and “No” indicate failure and success of amplification.

^d^ “-” indicates PCR amplification was not performed.

**Table 3:** **Known *Puroindoline-D1 *alleles in bread wheat**

***Pina-D1***	***Pinb-D1***	**Phenotype**	**NCBI No.**	**Molecular characterization**	**References**
*Pina-D1a*	*Pinb-D1a*	Soft	DQ363911	Wild type	[2]
*Pina-D1b*	*Pinb-D1a*	Hard	AB262660	15,380-bp deletion	[29]
*Pina-D1k*	*-*	Hard	-	≈ 33-kb deletion	In this paper; [6]
*Pina-D1l*	*Pinb-D1a*	Hard	-	ORF shift: C deletion at position 265 of Pinb	[8,18]
*Pina-D1m*	*Pinb-D1a*	Hard	EF620907	Pro-35 → Ser	[18]
*Pina-D1n*	*Pinb-D1a*	Hard	EF620908	Trp-43 → Stop codon	[18]
*Pina-D1p*	*Pinb-D1a*	Hard	AY599893	Val13 → Glu	[20]
*Pina-D1q*	*Pinb-D1a*	Hard	AB181238	Asn-139 → Lys; Ile-140 → -Leu	[20]
*Pina-D1r*	*Pinb-D1a*	Hard	HM572327	10,415-bp deletion	[30]
*Pina-D1s*	*Pinb-D1a*	Hard	-	4,422-bp deletion	In this paper
*Pina-D1t*	*Pinb-D1a*	Hard	JN680739	Trp41 → Stop codon	[38]
*Pina-D1u*	*Pinb-D1a*	Hard	-	6,460-bp deletion	In this paper
*Pina-D1a*	*Pinb-D1b*	Hard	DQ363914	Gly-46 → Ser	[3]
*Pina-D1a*	*Pinb-D1c*	Hard	-	Leu-60 → Pro	[9]
*Pina-D1a*	*Pinb-D1d*	Hard	-	Trp-44 → Arg	[9]
*Pina-D1a*	*Pinb-D1e*	Hard	-	Trp-39 → Stop codon	[10]
*Pina-D1a*	*Pinb-D1f*	Hard	-	Trp-44 → Stop codon	[10]
*Pina-D1a*	*Pinb-D1g*	Hard	-	Cys-56 → Stop codon	[10]
*Pina-D1a*	*Pinb-D1p*	Hard	AY581889	ORF shift: A deletion at position 210 of Pinb	[39]
*Pina-D1a*	*Pinb-D1q*	Hard	EF620909	Ser-44 → Leu	[34]
*Pina-D1a*	*Pinb-D1r*	Hard	AJ619022	ORF shift: G insertion at position 127	[40]
*Pina-D1a*	*Pinb-D1s*	Hard	AJ619021	ORF shift: G insertion and C → A at positions 127 and 205, respectively	[40]
*Pina-D1a*	*Pinb-D1t*	Hard	EF620910	Gly-47 → Arg	[18]
*Pina-D1a*	*Pinb-D1u*	Hard	EF620911	ORF shift: G deletion at position 126	[41]
*Pina-D1a*	*Pinb-D1v*	Hard	AY598029	Leu-9 → Ile	[20]
*Pina-D1a*	*Pinb-D1w*	Hard	AY640304	Pro-114 → Ile	[20]
*Pina-D1a*	*Pinb-D1x*	Hard	AM909618	C to A at position 257 and Gln-99 → stop codon	[42]
*Pina-D1a*	*Pinb-D1aa*	Hard	EF620912	ORF shift: C to A at position 96 and A deletion at position 210	[19]
*Pina-D1a*	*Pinb-D1ab*	Hard	AB302894	Gln-99 → Stop codon	[43]
*Pina-D1a*	*Pinb-D1ac*	Hard	-	G to T at position 257 and Gln-99 → stop codon	[44]

However, there were still four cultivars remaining after the screening with the new marker *Pina-N3*, i.e. one landrace cultivar Zuantoubaike with the absence of a *Pina* gene and three landrace cultivars Yumai, Changyaomai and Yangqingke with an absence of *Pina* and *Pinb* genes. Results from the primer walking strategy showed that amplicons could be obtained with primer sets through Pina-1 to Pina-9 and Pina-16 to Pina-25 whereas no targeted fragment was amplified with primer sets Pina-10 through Pina-15 in the Chinese landrace cultivar Zuantoubaike (Table 2). A new molecular marker *Pina-N4* spanning deletion fragment was developed for identifying this new allele, and then a 524-bp fragment was successfully obtained in Zuantoubaike while it could not amplify a targeted band in other cultivars (Figure 2F). Sequencing results of the 524-bp fragment indicated a 6,460-bp deletion from −4435 bp to 2024 (reference to ATG of the *Pina*) in Zuantoubaike when compared with the *Ha* sequence on the chromosome of 5DS in Chinese Spring (NCBI No: CT009735). This mutation with a single 6,460 bp deletion of *Pina* gene could be designated as *Pina-D1u* (Table 3) according to the above-mentioned nomenclature.

For four cultivars without *Pina* and *Pinb* genes, including one Netherlands wheat cultivar Pcatan and three Chinese landraces Yumai, Changyaomai and Yangqingke, expected fragment sizes could only be gained in primer sets through Pina-1 to 3 and Pinb-9 to Pinb-13. Therefore, an approximate 33-kb deletion fragment containing *Pina* and *Pinb* coding regions could be deduced to occur in those three landraces when compared with the *Ha* sequence on the chromosome of 5DS in Chinese Spring (Table 3, Figure 1). However, a valid marker spanning this big deletion for specifying the location of this new allele was not obtained due to high similarity with the *Ha* loci of A and B genome in this region, even though several primer sets spanning this deletion were designed. This mutation with a single ≈ 33-kb deletion containing *Pina* and *Pinb* coding regions was temporarily designated as *Pina-null/Pinb-null* due to the large deletion simultaneously related to *Pina* and *Pinb* genes. In previous reports [6,45], some cultivars had been found to lack the *Pina* and *Pinb* coding regions, designated *Pina-D1k* by Morris and Bhave [13]. However, *Pina-null/Pinb-null* is still used for describing this allele in this study because it is not known if the above four cultivars have the same molecular characterization on the DNA level with *Pina-D1k* allele.

### Distribution of *Puroindoline-D1* alleles and their association with grain texture

Amongst hard cultivars from different countries, Chinese landraces showed the highest diversity on *Puroindoline-D1* genes and possessed 11 types of *Puroindoline-D1* alleles in hard wheat landraces (Table 4). CIMMYT hard wheat, only composed of two kinds of *Puroindoline-D1* alleles, showed the lowest diversity among the four countries surveyed and *Pina-D1b* was predominant with the high percentage of 94.6%, which is consistent with previous studies [14,17]. In wheat cultivars from Australia and Netherlands, *Pinb-D1b* was predominant with the high percentage of 73.6% and 56.7%, respectively. In Chile, *Pina-D1b* and *Pinb-D1b* are almost equally distributed in hard wheat cultivars. Surprisingly, 4 out of 6 cultivars with scarce allele *Pinb-D1d* showing relative superior processing quality [15] was found in Netherlands. *Pinb-D1p* was only found, and prevalent, in Chinese landraces (Table 4). Notably, based on five kernels’ results, one Chinese landrace cultivar, Bailaolaibian, possesses a double mutation genotype *Pina-D1r/Pinb-D1p* and its SKCS hardness index is 73.2.

**Table 4:** **Distribution of *Puroindoline-D1 *alleles in bread wheat from China, Mexico, Australia, Chile and Netherlands**

	**Phenotype**	**China**	**Mexico**	**Australia**	**Chile**	**Netherlands**	**Total**
**Sample No.**	**-**	**204**	**104**	**88**	**51**	**44**	**493**
*Pina-D1a/Pinb-D1a*	Soft	80	10	19	16	14	139
*Pina-D1b/Pinb-D1a*	Hard	0	89	17	19	4	129
*Pina-D1l/Pinb-D1a*	Hard	3	0	0	0	0	3
*Pina-D1n/Pinb-D1a*	Hard	4	0	0	0	0	4
*Pina-D1r/Pinb-D1a*	Hard	21	0	0	0	0	21
*Pina-D1s/Pinb-D1a*	Hard	28	0	0	0	0	28
*Pina-D1u/Pinb-D1a*	Hard	1	0	0	0	0	1
*Pina-D1a/Pinb-D1b*	Hard	15	5	51	17	17	105
*Pina-D1a/Pinb-D1c*	Hard	0	0	0	0	4	4
*Pina-D1a/Pinb-D1d*	Hard	0	0	1	1	4	6
*Pina-D1a/Pinb-D1p*	Hard	46	0	0	0	0	46
*Pina-D1a/Pinb-D1q*	Hard	1	0	0	0	0	1
*Pina-D1a/Pinb-D1t*	Hard	1	0	0	0	0	1
*Pina-D1r/Pinb-D1p*	Hard	1	0	0	0	0	1
*Pina-null/Pinb-null*	Hard	3	0	0	0	1	4

In this study, we divided all cultivars surveyed into two groups of Chinese landraces and introduced cultivars for analyzing the association of *Puroindoline-D1* alleles with grain texture due to the obvious difference on agronomic traits between them. A two-year average of SKCS hardness index was compared by significant differences of variance analysis among different genotypes in Chinese landraces and introduced cultivars even though grain texture possessed a high heritability of more than 80% based on previous reports. In Chinese landraces, the cultivars with *Pina-null/Pinb-null* allele possess the highest SKCS hardness index among several genotypes (Table 5). Due to the absence of both *Pina-D1* and *Pinb-D1* genes, those cultivars have a similar grain texture to durum wheat which also has an extremely high SKCS hardness index. Three types of *Pina-D1* mutations resulting in PINA protein null do not show significant difference of SKCS hardness but they all have significantly higher SKCS hardness than *Pinb-D1b* genotype (Table 5). Of the introduced cultivars, PINA-null and PINB-D1c genotypes show significantly higher SKCS hardness than PINB-D1b and PINB-D1d genotypes, which are consistent with the results of Morris et al. [10], that PINB-D1c genotype possesses significantly higher SKCS hardness than PINB-D1b genotype.

**Table 5:** **Association of *Puroindoline-D1 *alleles with SKCS hardness in bread wheat**

		**Chinese landraces**		**Introduced cultivars**		**NILs**	
**Protein**	**Genotype**	**Sample No.**	**SKCS ± SD**	**Sample No.**	**SKCS ± SD**	**Sample No.**	**SKCS**
PINA-D1a/PINB-D1a	*Pina-D1a/Pinb-D1a*	80	38.8c ± 7.06	59	34.8d ± 8.25		
PINA-null/PINB-D1a	*Pina-D1b/Pinb-D1a*			129	85.2a ± 8.20	1	86.8a
	*Pina-D1l/Pinb-D1a*	3	81.9a ± 7.4				
	*Pina-D1n/Pinb-D1a*	4					
	*Pina-null/Pinb-null*	3					
	*Pina-D1r/Pinb-D1a*	21					
	*Pina-D1s/Pinb-D1a*	28					
PINA-D1a/PINB-D1b	*Pina-D1a/Pinb-D1b*	15	73.6b ± 12.51	90	78.4c ± 7.41	1	69.4c
PINA-D1a/PINB-D1c	*Pina-D1a/Pinb-D1c*			4	88.1a ± 5.63	1	77.3b
PINA-D1a/PINB-D1d	*Pina-D1a/Pinb-D1d*			6	80.0bc ± 5.78	1	69.8c
PINA-D1a/PINB-D1e	*Pina-D1a/Pinb-D1e*					1	74.3b
PINA-D1a/PINB-D1f	*Pina-D1a/Pinb-D1f*					1	77.8b
PINA-D1a/PINB-D1g	*Pina-D1a/Pinb-D1g*					1	76.8b
PINA-D1a/PINB-D1p	*Pina-D1a/Pinb-D1p*	46	77.2b ± 7.26				
	Total	200	63.7	288	72.6		

Different letters indicated a significant difference at the 5% level.

In order to further investigate the influence of *Puroindoline-D1* alleles on grain texture and obtain a clean association of *Puroindoline-D1* alleles with SKCS hardness without impact of other loci in the genome, seven near-isogenic lines with different *Puroindoline-D1* alleles were used to compare their SKCS hardness index (Table 5). Results indicate that PINA-null genotype possesses the significantly highest SKCS hardness, whereas PINB-D1b and PINB-D1d genotypes possess the significantly lowest SKCS hardness amongst seven different hard genotypes (Table 5). These results are consistent with above-mentioned results derived from Chinese landrace cultivar and introduced wheat cultivars.

## Discussion

Grain texture, which is mainly controlled by the *Puroindoline-D1* genes on the 5DS chromosome, has an important impact on the milling and processing qualities of bread wheat (*Triticum aestivum* L.). It has shown mutations in either *Pina-D1* or *Pinb-D1* allele result in a hard endosperm in bread wheat based on discoveries of many *Puroindoline-D1* alleles. However, most of the mutations identified previously in bread wheat resulted from a single nucleotide polymorphism (SNP) in *Pinb-D1* or *Pina-D1* genes. In this study, we found that diverse mutations occurred in the *Ha* loci of bread wheat, in the form of large deletions including entire or partial *Pina-D1* coding region, and caused the PINA-null allele. In the long term, the lack of a straightforward marker for identifying the PINA-null allele leads us to develop a *Pina-N1* marker for detection of *Pina-D1b* allele [29].

The previous most common approach for detecting *Pina-D1b* allele was to examine the presence or absence of the PINA protein, however this approach fails to identify the status of PINA-null allele at the DNA level. Therefore, almost all of PINA-null allele was taken into account as *Pina-D1b* allele [17,18,39,46]. However, the findings from our study show the PINA-null allele to possess a completely different molecular characterization at the DNA level. The PINA-null (*Pina-D1b* previously called) allele is known to be the most prevalent genotype in the CIMMYT bread wheat cultivars [14,17]. In this study, all of PINA-null allele in CIMMYT wheat surveyed was shown to have the *Pina-D1b* allele. The four molecular markers (*Pina-N1* and *Pina-N2* we previously developed; *Pina-N3* and *Pina-N4* in this study) will be useful for straightforward and efficient identification of PINA-null alleles in bread wheat cultivars.

Up to now, many *Pina-D1* and *Pinb-D1* alleles have been identified in different geographic bread wheat cultivars from around the world. Amongst different countries or regions, that China seems to possess a relatively more diverse germplasm of bread wheat on the genotype of grain texture, based on several investigations of *Puroindoline-D1* alleles [10,17,18,47] because almost all of the *Puroindoline-D1* alleles previously reported in other countries or regions, outside of China, have been aslo found in Chinese wheat cultivars whereas a number of *Puroindoline-D1* alleles appear to be exclusive to Chinese wheat cultivars so far, e.g. *Pinb-D1p*, *Pinb-D1q*, *Pinb-D1t*, *Pinb-D1u*, *Pinb-D1v*, *Pinb-D1w*, *Pinb-D1x*, *Pinb-D1aa*, *Pinb-D1ac*, *Pina-D1m*, *Pina-D1n*, *Pina-D1p*, *Pina-D1q, Pina-D1r* etc. [18,20,34,41,42,44]. In this study, Chinese landraces also showed the highest diversity of *Puroindoline-D1* alleles among wheat cultivars from five different countries. Due to the PINA-null allele, which possibly result from an evolution of hexaploid wheats from *Ae. tauschii*[30,31], the molecular mechanism of each cultivar with PINA-null allele has been illustrated by either known molecular markers or the primer walking strategy. The discovery of *Pina-D1v* and *Pina-D1u* showed that five types of PINA-null alleles (*Pina-D1b*, *Pina-D1s*, *Pina-D1r*, *Pina-D1v* and *Pina-D1u*) have different deletion sites from each other, suggesting that the deletions could have occurred independently. According to previous reports [1,7,10,22] and recent work on *Puroindoline-D1* genes in *Ae. tauschii* (Personal communicate with Craig F. Morris in Washington State University), all five of the above-mentioned *Pina-D1* alleles are not found in *Ae. tauschii* so far, suggesting that the big deletions of the above *Pina-D1* allele possibly occurred during the formation of hexaploid wheat. Interestingly, all wheat cultivars with the PINA-null allele from CIMMYT, Australia, Netherlands and Chile are further identified as *Pina-D1b* allele in this study, whereas Chinese landraces with PINA-null allele are shown to possess four different alleles of *Pina-D1s*, *Pina-D1r*, *Pina-D1v* and *Pina-D1u*, which is possibly because China is the secondary origin center of hexaploid wheat in the word.

The Yellow and Huai valley is the largest and the most important Chinese wheat production region and is greatly responsible for the national food security guarantee. However, wheat production and quality have not significantly improved during the past decade in this region. A potential reason for this is mainly because the narrow genetic basis of modern wheat cultivars is a serious obstacle against sustaining and improving wheat productivity due to rapid vulnerability of genetically uniform cultivars by potentially new biotic and abiotic stresses. In an attempt to improve the status quo, a large number of alien wheat germplasms were introduced to the Yellow and Huai wheat production region of China. Even though we have previously reported the wheat cultivars of the Yellow and Huai wheat production region regarding *Puroindoline-D1* alleles [30], investigation in this study primarily focused on landraces and introduced cultivars that are or were being core parents during the breeding process in the Yellow and Huai wheat production region. The cultivars previously used in Chen et al. [30] were mainly historical cultivars and modern cultivars, and all accessions we used previously were excluded in this study. Therefore, the work carried out in current and previous [30] studies could provide a more comprehensive understanding of wheat germplasms, particularly as potential parents for wheat breeding programs in view of grain texture in the Yellow and Huai wheat production region.

## Conclusion

In the present study, molecular characterization of the *Puroindoline-D1* allele was investigated in bread wheat cultivars from five geographic regions. Two novel alleles *Pina-D1s* and *Pina-D1u* at the *Pina-D1* locus were characterized at the DNA level by a primer walking strategy, and corresponding molecular markers were developed for straightforward identification of these two alleles. Analysis of the association of *Puroindoline-D1* alleles with grain texture indicated that wheat cultivars with *Pina-null/Pinb-null* allele have the highest SKCS hardness index amongst the different genotypes, and wheat cultivars with the PINA-null allele have significantly higher SKCS hardness index than those of *Pinb-D1b* and *Pinb-D1p* alleles.

## Abbreviations

SKCS: Single kernel characterization system; Ha: Hardness; Pina-D1: *Puroindoline a-D1*; Pinb-D1: *Puroindoline b-D1*; Gsp-1: Grain Softness Protein; CIMMYT: International maize and wheat improvement center; dCAPS: derived Cleaved Amplified Polymorphic Sequences; PCR: Polymerase chain reaction; SNP: Single nucleotide polymorphism; NIL: Near-isogenic line.

## Competing interests

The authors declare that they have no competing interests.

## Authors’ contributions

FC and DC designed and prepared the manuscript. HL and FC performed identification of phenotypes and genotypes and participated in primer walking strategy. All authors read and approved the final manuscript.
